# Variability in CRP, regulatory T cells and effector T cells over time in gynaecological cancer patients: a study of potential oscillatory behaviour and correlations

**DOI:** 10.1186/1479-5876-12-179

**Published:** 2014-06-23

**Authors:** Mutsa T Madondo, Sandra Tuyaerts, Brit B Turnbull, Anke Vanderstraeten, Holbrook Kohrt, Balasubramanian Narasimhan, Frederic Amant, Michael Quinn, Magdalena Plebanski

**Affiliations:** 1 Department of Immunology, Vaccine and Infectious Diseases Laboratory, Monash University, Melbourne, Australia; 2 Division of Gynaecologic Oncology, Department of Oncology, KU Leuven, Leuven, Belgium; 3 Department of Statistics and Department of Health Research and Policy, Stanford University, Stanford, CA, USA; 4 Division of Haematology and Oncology, Centre for Clinical Sciences Research, Stanford University, Stanford, CA, USA; 5 Womens Cancer Research Centre, Royal Women’s Hospital, Melbourne, Australia; 6 Department of Immunology, Vaccine and Infectious Diseases Laboratory, School of Medicine and Health Science, Monash University Alfred Hospital Campus, 99 Commercial Road, Prahran, VIC 3181, Australia

**Keywords:** Inflammation, CRP, Gynaecological malignancies, Treg, Teff

## Abstract

**Background:**

The inflammatory marker, C reactive protein has been proposed to also be a biomarker for adaptive immune responses in cancer patients with a possible application in time based chemotherapy. Fluxes in serum CRP levels were suggested to be indicative of a cyclical process in which, immune activation is followed by auto-regulating immune suppression. The applicability of CRP as a biomarker for regulatory or effector T cells was therefore investigated in a cohort of patients with gynaecological malignancies.

**Methods:**

Peripheral blood samples were obtained from a cohort of patients at 7 time points over a period of 12 days. Serum and mononuclear cells were isolated and CRP levels in serum were detected using ELISA while regulatory and effector T cell frequencies were assessed using flow cytometry. To test periodicity, periodogram analysis of data was employed while Pearson correlation and the Wilcoxon signed rank test were used to determine correlations.

**Results:**

The statistical analysis used showed no evidence of periodic oscillation in either serum CRP concentrations or T_eff_ and T_reg_ frequencies. Furthermore, there was no apparent correlation between serum CRP concentrations and the corresponding frequencies of T_regs_ or T_effs_. Relative to healthy individuals, the disease state in the patients neither significantly affected the mean frequency of T_regs_ nor the mean coefficient of variation within the T_reg_ population over time. However, both T_eff_ mean frequency and mean coefficient of variation were significantly reduced in patients.

**Conclusion:**

Using our methods we were unable to detect CRP oscillations that could be used as a consistent serial biomarker for time based chemotherapy.

## Background

The link between inflammation and tumour development has been widely reported and recently, in an update to their seminal paper on the hallmarks of cancer, Hanahan and Weinberg refer to inflammation as an “enabling characteristic” for tumour development [1]. This link is well illustrated in gynaecological malignancies. The processes of ovulation and menstruation are associated with inflammation of the ovarian surface and endometrial epithelia [2,3]. During ovulation there is an increase in the macrophage population in the perifollicular stroma of pre-ovulatory follicles [4,5]. These macrophages produce pro-inflammatory cytokines Interleukin (IL)-6, IL-1β and Tumour Necrosis Factor (TNF), [2]. Oestrogen in the endometrium also induces the up-regulation of these pro-inflammatory cytokines [6]. Over time, the cyclical process of epithelial damage and repair, involving inflammation, predisposes to neoplastic growth. Not only does inflammation play a role in tumourigenesis, it also facilitates disease progression. The tumour microenvironment is rich in inflammatory mediators produced by tumour cells and infiltrating leukocytes [7-9]. Specifically, IL-6 triggers tumour cells to produce matrix metalloproteinase (MMP)-9 that promotes tumour growth by initiating angiogenesis [10].

C reactive protein (CRP), an acute phase protein produced by hepatocytes is the most widely used indicator of inflammation. The transcription of CRP is induced by IL-6 alone while the presence of either IL-1β or TNF augments its production [11,12]. Gynaecological cancer patients exhibit elevated levels of CRP, however these levels are not static. In a preliminary study of melanoma and ovarian cancer patients, serum CRP levels oscillated about a mean with a periodicity (λ) of 7 days [13]. The dynamic state of inflammation could indicate an underlying persistent but regulated anti-tumour immune response in the cancer patients, which is characterised by a cyclical repeating process of endogenous auto-vaccination, preceding suppression by regulatory cells.

In the past few decades the role of the adaptive immune system in driving anti-tumour immunity has been highlighted. Studies have shown a positive correlation between the number of tumour infiltrating effector T cells and patient survival, as well as a negative correlation with Foxp3^+^CD25^Hi^CD4^+^ regulatory T cells [14,15]. Inflammatory cytokines and chemokines such as TNF and CCL22 facilitate the infiltration of T cells into the tumour microenvironment. When activated, effector T cells up-regulate CD25 and transiently express FoxP3 [16]. This enables them to expand and secrete pro-inflammatory cytokines. FoxP3^+^CD25^Hi^CD4^+^ regulatory T cells, which express high levels of the CCL22 receptor CCR4, also infiltrate tumour sites where they expand in response to TNF and IL-2 produced by effector T cells, and subsequently inhibit the immune response [17-19].

A lag between the expansion phase of effector T cell and regulatory T cell populations could account for the oscillations in CRP levels observed in cancer patients. If this is the case, oscillations in serum CRP levels could be used to target regulatory T cells for depletion during their expansion phase, by using chemotherapy. Pre-treatment results are reported herein on a prospective phase II trial of cyclophosphamide treatment for gynaecological malignancy, where repeated sampling was taken prior to trial initiation. This allowed the opportunity to assess the hypothesis that CRP levels oscillate, and may be indicative of effector and regulatory T cell proportions and that the latter may also display predictable oscillations in patients with advanced gynaecological malignancies.

## Methods

### Trial design and patient details

In total, 19 patients with gynaecological tumours were included in this bicentric phase II trial. The patients had end stage treatment-refractory gynaecological tumours, i.e. ovarian cancer (n = 5), papillary serous cystadenocarcinoma (n = 9), adenocarcinoma (n = 1), serous cystadenocarcinoma (n = 1), endometrial carcinoma (n = 1), peritoneal tumour, (n = 1) or malignant mixed müllerian tumours (n = 1) and were recruited from the Royal Women’s Hospital, Melbourne, Australia (n = 12) and the University Hospital Leuven, Belgium (n = 7). Patient age ranged from 43–82 years (Table 1). The relative institution’s research and ethics committees approved the study. From the time of recruitment, written consent was obtained from patients and 7 blood collections were performed over 12 days with daily and second daily bleeds, to assess the presence of a CRP cycle. Blood was collected in the morning on days 1, 3, 5, 6, 8, 10 and 12 with one exception who had blood collected on days 1, 2, 4, 5, 6, 8, 12. Following collection, the blood samples were immediately transported to the laboratory for processing with a maximum of 12 hours between blood collection and freezing of plasma. Based on predicted CRP cycling patterns [13], patients went on to received treatment with low-dose cyclophosphamide, 50 mg administered p.o. twice daily for 3 consecutive days. Blood was also collected from 7 healthy volunteers at the Alfred Hospital, Melbourne, Australia. Ethics was obtained from the institution’s research board and written consent was also obtained from the volunteers. The blood collection protocol for the healthy volunteers was similar to that of the cancer patients.

**Table 1:** Patient summary

**Patient ID**	**Age**	**Tumour type**	**Prior treatment**	**Disease status at inclusion**
IRS 11	70	Papillary serous cystadenocarcinoma	Surgery, 2 lines chemo,	Progessive disease (PD) with rising CA125
IRS 12	79	Papillary serous cystadenocarcinoma	Surgery, 2 lines chemo,	PD – with progressive nodal abnormalities above and below diaphragm and new peritoneal abdo/pelvic disease
IRS 13	69	Papillary serous cystadenocarcinoma	Surgery, 2 lines chemo,	PD with short bowel syndrome
BLV 01	74	Ovarian carcinoma	Surgery, 3 lines chemo	PD with newly formed hepatic lesion, hepatogastric, lymph node, peritoneal and mesenteric lesions present
BLV 02	80	Ovarian carcinoma	Surgery, 3 lines chemo, provera	PD with increase of peritoneal metastases with ascites formation
BLV 03	73	Ovarian carcinoma	3x surgery, 4 lines chemo	PD with increase and new formation of abdominal metastases and possible lymph node metastases
BLV 04	64	Peritoneal tumour	Surgery, 6 lines chemo, avastin	PD with peritoneal, omental, vertebral, hepatic and possible gastric metastases
BLV 05	62	Endometrial carcinoma	2x surgery, 1 line chemo, radiotherapy	PD with increase of hepatic, brain and possible renal metastases
BLV 06	74	Malignant mixed Müllerian tumour (MMMT)	Surgery, 1 line chemo	PD with increase of known retroperitoneal lesions and occurrence of a new lesion
BLV 07	82	Ovarian carcinoma	2x surgery, 5 lines chemo, provera	PD with increase of LN metastases in upper abdomen, metastases in mediastinum and formation of lung metastases
IRS 01	43	Adenocarcinoma	Surgery, 1 line chemo, radiotherapy and EGFR inhibitor	PD with recurrence in supraclavicular fossa lymph node
IRS 02	45	Ovarian carcinoma	Surgery, 3 lines chemo, radiotherapy	PD with hepatic involvement
IRS 03	54	Papillary serous cystadenocarcinoma	Surgery, 2 lines chemo, radiotherapy	PD with rising CA-125 marker
IRS 04	63	Papillary serous cystadenocarcinoma	Surgery, 5 lines chemo, hormone therapy	PD with rising CA-125 marker
IRS 05	68	Serous cystadenocarcinoma	2x Surgery, 1 line chemo, hormone therapy	PD with rising CA-125 marker
IRS 06	74	Papillary serous cystadenocarcinoma	Surgery, 2 lines chemo	PD rising CA-125 marker, PAN and adrenal gland involvement
IRS 07	66	Papillary serous cystadenocarcinoma	Surgery, 3 line chemo, radiotherapy, 1x EGFR inhibitor	PD with rising CA125
IRS 08	63	Papillary serous cystadenocarcinoma	Surgery, 2 lines chemo	PD with rising CA125 level
IRS 09	63	Papillary serous cystadenocarcinoma	Surgery, 5 lines chemo	PD with omental mass increased in size

### hsCRP plasma level measurements

Plasma was isolated from whole blood collected either in EDTA-coated tubes (Belgian patients) or in serum separation tubes (Australian patients) by centrifugation. After removing cellular and protein debris, plasma was aliquoted and stored at −80°C for later use. Hs CRP levels were determined by ELISA (Human High Sensitivity C-Reactive Protein ELISA kit; Cusabio Biotech Co., LTD, China) according to manufacturer’s instructions. For each treatment cycle, batch analysis was performed on all the necessary samples. To increase experimental precision, all samples were analysed in duplicate.

### Peripheral blood mononuclear cells isolation

Mononuclear cells were obtained from peripheral whole blood collected in EDTA-coated tubes via Ficoll (Amersham Pharmacia Biotech, Sweden) density gradient centrifugation. The isolated PBMCs were cryopreserved using a freeze mixture containing 10% DMSO (Sigma-Aldrich, Australia) and either 90% Foetal Calf Serum (Australian samples, JRH Bioscience) or human AB serum (Belgian samples, Sera Laboratories International) and stored in freezing containers (Nalgene) and finally in liquid nitrogen until use. For use, cells were thawed in a 37°C water-bath and quickly re-suspended using AIM-V media (Invitrogen) with 5% human serum (Sigma). PBMC samples were available at all time points from 10 patients in total.

### Flow cytometric analysis

To determine the frequency and phenotype of T cell populations in PBMCs of patients with gynaecological malignancies and age matched female, healthy volunteers, multicolour flow cytometric analysis was performed using the following surface antibodies: anti-CD3 Q655 (Invitrogen), anti-CD4 AF700 (BD Pharmingen), anti-CD25 PE (BD Pharmingen), and anti-CD127 Biotin (BD Pharmingen). Following primary staining, a fixable dead cell dye (Invitrogen) was also used to distinguish between dead and live cells. Intracellular levels of FoxP3 were determined following fixation and permeabilization using a fixation/permeabilization buffer kit (eBioscience) then staining with anti-FoxP3 PercpCy5.5 (eBioscience). Flow cytometry data was acquired on a Becton Dickinson LSR II using FACSDiva software, collecting a minimum of 100,000 events per sample. Fluorescence minus one (FMO) and isotype matched antibodies were used as controls with all samples. Data were analysed using Flowjo software (TreeStar).

### Serum cytokine analysis

To assess serum IL-6 concentration, frozen sera from all patients were thawed over night at 4°C. BD™ Cytometric bead array was used for IL-6 (BD) detection in 50 μl of undiluted serum following the instructions prescribed by the manufacturer. Samples were then acquired by Flow cytometry on a Becton Dickinson LSR II using FACSDiva software, collecting a maximum of 5,000 events per sample. Data were analysed using Flowjo software (TreeStar).

### Statistics

To determine the respective periodicity of serum CRP concentration, and Teff and Treg frequencies periodogram analysis was used. The null hypothesis was that there was no consistent period to the measurements, which implies no peaks in the mean periodogram beyond noise. Individual subjects’ periodograms were calculated and standardized to have sum of squares equal to 1, then averaged pointwise. Under the null hypothesis, the population pointwise mean periodogram is a horizontal line at 1. The pointwise, lower one-sided 95% confidence bound for the mean periodogram was calculated by the bias-corrected bootstrap method. This lower confidence bound was then compared to the null mark at 1. Exceeding 1 at a peak would be necessary to suggest a significant peak.

The Pearson correlation coefficient between T_eff_ first differences and CRP first differences was estimated for each subject, and the Wilcoxon signed rank test was used to test whether the mean correlation coefficient was non-zero. The same procedure was performed to test for a relationship between T_reg_ and CRP, T_regs_ and T_effs_, and CRP and IL-6. The first difference is defined as the change in value from one time point to the next.

Coefficients of variation in the frequencies of T_regs_ and T_effs_ among 7 time points over a period of 12 days were determined by initially calculating the standard deviation, which was then expressed as percentages of the mean frequency of the respective population over the 7 time points. Statistical significances between mean values of T_reg_ and T_eff_ frequencies as well as coefficients of variation for both populations were calculated using non-parametric (Wilcoxon Mann Whitney) tests. P values < 0.05 were considered significant. All mean values were presented ± the standard error from the mean (SEM).

## Results

### CRP levels, and T_reg_ and T_eff_ frequencies did not appear to be oscillatory

**Figure 1:**
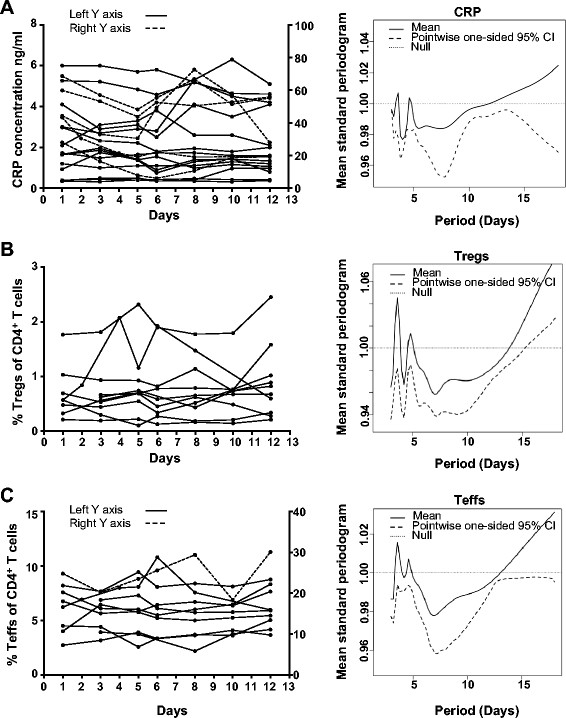
**The periodicity of serum CRP concentration, and T**_**reg **_**and T**_**eff **_**frequency.** Patients with gynaecological malignancies had blood collected 7 times over a period of 12 days. Serum was collected from 19 patients. While in 10 of the patients, PBMCs were also isolated. CRP levels in sera were determined along with the frequencies of Tregs and Teff within CD4^+^ T cells from PBMCs. The values for **A**, serum CRP concentration (n = 19, solid lines use the left Y axis while dashed lines use the right Y axis) (mg/L), **B** Treg and **C**, Teff frequencies (%, n = 10, solid lines use the left Y axis while dashed lines use the right Y axis) over time (days) were plotted in spaghetti plots. The periodicity for **A**, CRP concentration, **B**, Treg and **C**, Teff frequencies were assessed using the null hypothesis, that the population pointwise mean periodogram was a horizontal line at 1 (dotted line). The pointwise lower one-sided 95% confidence bound (dashed line) needed to exceed the null mark (dotted line) to suggest a significant peak.

The oscillation in serum CRP concentration with a period of 7 days has previously been reported in 15 late-stage advanced melanoma and 4 late-stage advanced ovarian cancer patients [13]. In a cohort of 19 patients with gynaecological malignancies, we investigated the periodicity of serum CRP concentration oscillations over time (raw data provided in Additional file 1: Table S1). In the individual patients, serum CRP concentrations appeared to fluctuate over time (Figure 1A), however when a mean standard periodogram analysis was carried out for the pooled patient cohort at every time point, while small peaks were apparent in the periodograms at 3.6 days and 4.6 days, neither was significant (Figure 1A). In a smaller cohort of 10 patients from whom PBMCs were collected, the periodicity of peripheral blood T_reg_ and T_eff_ frequencies was also investigated. T_regs_ were defined as expressing high levels of CD25 and FoxP3, which we confirmed expressed low levels of CD127, while T_effs_ were defined as expressing intermediate levels of CD25 (Additional file 2: Figure S1) as per previous studies [20,21]. Similar to serum CRP concentration, both frequencies appeared to fluctuate over time, in some individual patients (Figure 1B and C). In the pooled cohort, mean standard periodogram analysis revealed small peaks for T_reg_ frequencies at 4.8 days and for T_eff_ frequencies at 3.6 and 4.6 days, but none was significant (Figure 1B and C) statistically at the p = 0.05 level.

### Serum CRP concentrations were not correlated with T_reg_ or T_eff_ frequencies

Although there was no evidence of a predictable oscillatory pattern of serum CRP concentration over time, we investigated whether CRP could still be used as a surrogate marker for T_reg_ or T_eff_ frequency in peripheral blood. Using the 10 patients from which, both serum and PBMCs were collected, serum CRP concentrations from each time point within each individual patient were then correlated with serum IL-6 concentration as well as the corresponding frequencies of T_regs_ or T_effs_. The correlation coefficients from the patients showed a significant positive correlation between serum CRP and serum IL-6 concentrations (p = 0.03; Table 2). However, there appeared to be no apparent relationship between serum CRP concentration, and T_reg_ or T_eff_ frequencies (Table 3 and Table 4). T_regs_ and T_effs_ frequencies on the other hand, correlated significantly with each other (p = 0.01; Table 5).

**Table 2:** Correlation coefficients of CRP vs IL-6

**Stem**	**Leaf**
−0	32210
0	11113344
0	5579

null hypothesis: correlation coefficient mean = 0.

V = 98.5 p = 0.03 (Wilcoxon signed rank test).

**Table 3:** Correlation coefficients of CRP vs Tregs

**Stem**	**Leaf**
−0	320
0	124
0	5788

null hypothesis: correlation coefficient mean = 0.

V = 45 p = 0.08 (Wilcoxon signed rank test).

**Table 4:** Correlation coefficients of CRP vs Teffs

**Stem**	**Leaf**
−0	6
−0	100
0	122
0	679

null hypothesis: correlation coefficient mean = 0.

V = 42 p = 0.16 (Wilcoxon signed rank test).

**Table 5:** Correlation coefficients of Tregs vs Teffs

**Stem**	**Leaf**
−0	32
0	44
0	56889
1	0

null hypothesis: correlation coefficient mean = 0.

V = 52 p = 0.01 (Wilcoxon signed rank test).

### Disease state in the cancer patients did not increase the variation of T_reg_ or T_eff_ frequencies within CD4^+^ T cells over time

**Figure 2:**
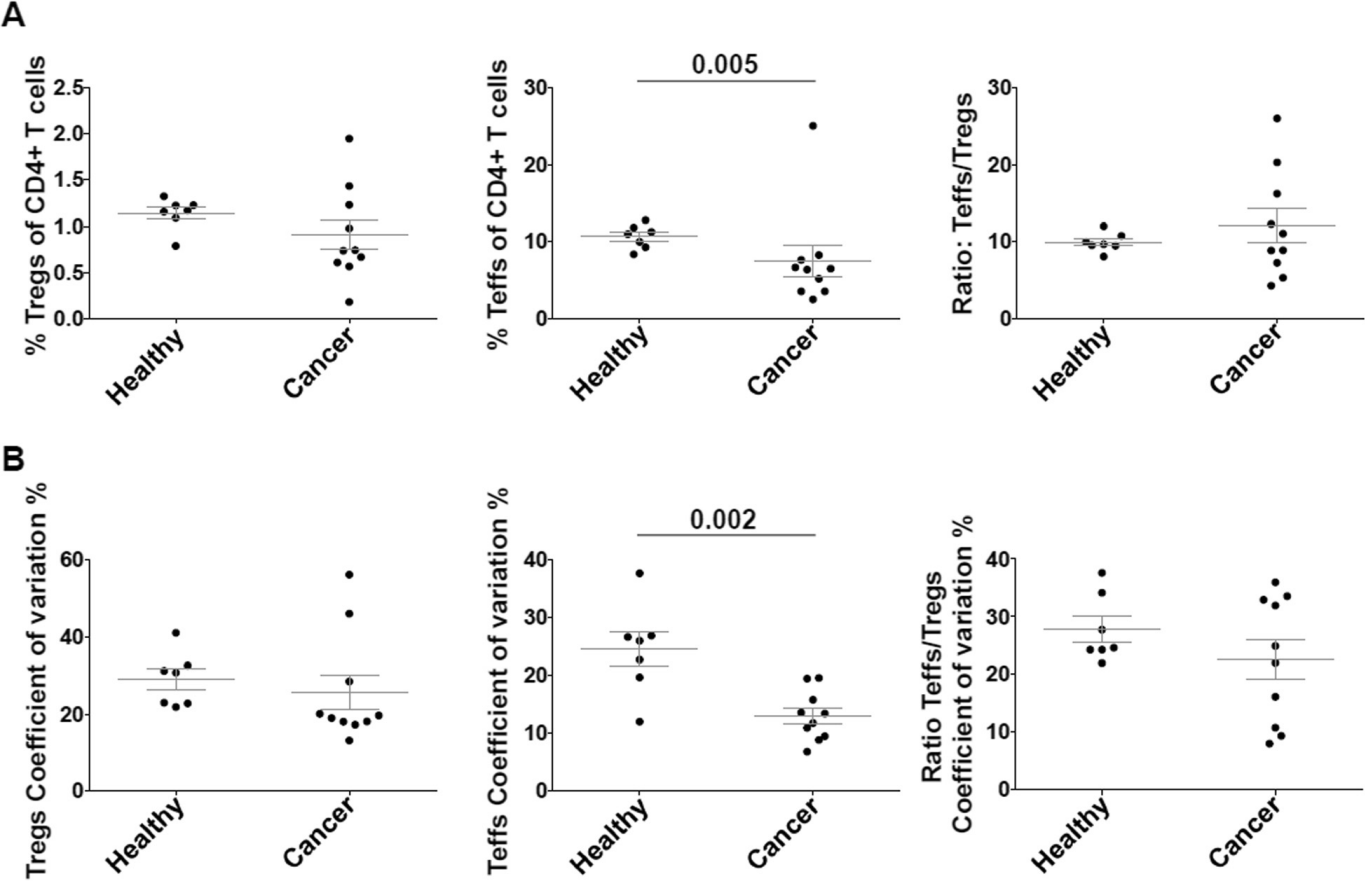
**Variation in T**_**reg **_**and T**_**eff **_**frequencies. A**, The mean frequency from 7 readings of T_regs_ and T_effs_ over a period of 12 days was obtained for each of 10 cancer patients and 7 healthy donors. The ratio of T_eff_ to T_regs_ was also calculated. **B**, The coefficients of variation in the frequencies of T_regs_ and T_effs_ as well as the ratio of T_effs_ to T_regs_ were determined by calculating the standard deviation among the 7 time points for each cancer patient and healthy donor and expressing it as a percentage of the mean frequency. Wilcoxon and Mann Whitney tests were used to determine significant differences between mean values, with p < 0.05 indicating significance. Graphs show mean ± standard error from the mean (SEM).

There did not appear to be a correlative linear relationship between inflammation as shown by serum CRP concentration and peripheral blood T_regs_ and T_effs_ frequencies. We investigated whether the disease state in patients may still influence the magnitude of variation in the frequencies of T_regs_ and T_effs_ over time. We initially compared the mean frequencies of T_regs_ and T_effs_ within CD4+ T cells between 10 cancer patients and 7, age and sex matched, healthy donors. Although the mean frequency of T_effs_ only, was significantly lower in cancer patients (8% ± 2%) than healthy donors (11% ± 0.6%, p = 0.005; Figure 2A), the mean ratio of T_effs_ to T_regs_ was not significantly different. We then assessed whether cancer patients, who have raised inflammation, evident by higher CRP levels relative to healthy individuals [13], exhibited greater variation in the frequencies of either T_regs_ or T_effs_ over time. The variation in T_reg_ and T_eff_ frequencies from 7 blood collections over a 12-day period was compared between the 7 healthy volunteers and 10 cancer patients. Both cancer patients and healthy volunteers had a similar mean coefficient of variation in T_reg_ frequency (26% ± 5% and 29% ± 3% respectively, Figure 2B). The mean coefficient of variation in T_effs_ frequency was significantly lower in cancer patients (13% ± 1%, p = 0.002) compared to healthy volunteers (22% ± 2%, Figure 2B). While the mean coefficient of variation in the ratio of T_effs_ to T_regs_ was lower in cancer patients compared to healthy volunteers (21.7% ± 3.3% and 27.8% ± 2.2%, Figure 2B), this was not significant.

## Discussion

The dynamic nature of the adaptive immune responses has been described in two ways. Firstly, there are diurnal fluctuations, regulated by the circadian clock exemplified in CD4 T cells by the rhythmic expression of genes that control cytokine secretion and cell function [22]. Secondly, antigen dependent fluctuations occur during acute infections and the kinetics of the immune response are controlled by a system of positive and negative feedback mechanisms designed to limit the immune response when pathogenic insults are resolved. In chronic diseases such as cancer, antigenic clearance does not occur and the persistent antigen exposure results in a constant state of immune activation. The tumour however, limits immune activation by secreting immune inhibitory cytokines such as IL-10, and tumour growth factor (TGF)-β as well as inducing regulatory cell populations including myeloid derived suppressor cells (MDSCs) and regulatory T cells [18,23-25]. Although intratumoural immune cells are skewed towards immune inhibition, there may still exist a homeostatic balance that needs to be maintained in the periphery of cancer patients in which an oscillating sequence of immune activation is followed by negative feedback immune suppression [13]. The study herein however, showed that CRP concentrations in all the patients did not appear to oscillate periodically nor did the concentration of CRP correlate significantly with T_reg_ or T_eff_ frequencies. Therefore, CRP may not be a practical useful surrogate marker for either cell population. In a study of 12 patients with melanoma, less than 50% showed possible time dependent CRP concentration profiles [26]. The two data sets available therefore suggest that inflammation in cancer patients may not consistently follow an oscillatory, sinusoidal (or other mathematical) pattern with a constant period or amplitude. This may be because any such model oversimplifies the inflammatory process, and correlations are easily disrupted by a number of potential additional *in vivo* co-parameters or process variations. Multiple factors influence the levels of cytokines that regulate inflammation. One such factor is the tumour growth. In patients with papillary thyroid cancer CD4^+^ T cell frequencies correlate with tumour size while T_reg_ frequencies correlate with lymph node metastasis [27]. Such variables may hence significantly influence the magnitude of T cell effector and suppressor frequency and function.

In our study, neither the coefficient of variation of both T_regs_ and T_effs_ nor that of the ratio of T_eff_ to T_regs_, in cancer patients was greater than that of healthy donors suggesting that in the periphery the fluctuation of T_reg_ within the CD4 population was not affected by the presence of the tumour. The coefficient of variation of T_effs_ was lower in cancer patients compared to healthy donors, which may have been due to immune suppression. Consistent with this suggestion, the frequency of T_effs_ was significantly lower in the patients than in healthy donors. Although T_reg_ frequencies in the periphery were not significantly increased, other cell subsets such as MDSCs may also facilitate immune suppression. Pro-inflammatory mediators have also been suggested to promote accumulation of MDSC in cancer patients’ peripheral blood [28]. Within the tumour microenvironment, the fluxes between T_reg_ and T_eff_ populations may be more evident.

Although changes in the frequencies of conventional T_reg_ and T_eff_ did not correlate with inflammation, it is still possible that minor subsets within each phenotype (T_reg_ or T_eff_) or their specific function over time, may correlate. Indeed, effector and regulatory T cells are heterogeneous populations of cells. Further breaking down these populations based on phenotype and function may also show greater variation between patients and healthy donors. For example, T_reg_ populations with enhanced suppressive function due to elevated expression of inhibitory receptors such as glucocorticoid induced tumour necrosis factor receptor (GITR) and cytotoxic T-lymphocyte antigen (CTLA)-4 as well as increased production of suppressive factors such as adenosine and cytokines TGF-β and IL-10, have been reported to be elevated in cancer patients [29-31]. Similarly, effector T cells can be broken down into different functional phenotypes such as IL-17 secreting and type-1 interferon secreting T_effs_. Type-1 interferon secreting T_effs_ promote proliferation of cytotoxic CD8 T cells, which contribute towards an anti-tumour effect [32-35]. It cannot therefore be excluded that the frequencies of some of these functional subsets, as well as CD8 T cells, may be subject to regulation by inflammatory factors, even when the results presented herein show that total T_reg_ and T_eff_ populations are not correlated to inflammatory status as reflected by CRP levels in blood. Mathematical models that aim to predict the balance that exists between immune activation and regulation within accessible blood samples, will therefore benefit from additionally taking into account the following variables: i) the effect of diurnal variation ii) the tumour growth rate iii) the heterogeneity present within both regulatory and effector T cell populations.

In conclusion, in our sample of patients with gynaecological malignancies, CRP concentrations do not oscillate in a consistent predictable manner, and do not correlate either positively or negatively with conventional T_reg_ or T_eff_ subsets. Therefore, there is no evidence to suggest that CRP can reliably be used across cancer patients as a surrogate, time-sensitive and most importantly, predictive marker, to reflect circulating effector or regulatory T cell frequencies, as previously suggested [13]. Time based therapy founded on modelling a consistent cyclical pattern of inflammation using serum CRP concentration as a predictive marker of regulatory T cell expansion may not be possible. However, we cannot exclude that further investigating the kinetics of inflammation in cancer patients, perhaps by taking more frequent blood samples or else by taking into consideration multiple inflammatory and immune-regulatory parameters, the progressive nature of immune suppression, as well as the heterogeneity of effector and regulatory T cell populations could all help in modelling more complex, and potentially predictive, equations.

## Abbreviations

T_regs_: Regulatory T cells; T_effs_: Effector T cells; CRP: C reactive protein.

## Competing interests

The authors declare that they have no competing interests.

## Authors’ contributions

FA, MQ and MP contributed to the design of the study and FA and MQ organised patient recruitment. MM, ST and AV conducted experiments, while MM, MP and ST analysed data and drafted the manuscript. HK, BT and BN carried out statistical analysis of data. All authors proofread, edited and approved the manuscript.

## Supplementary Material

Additional file 1: Table S1a CRP raw data**. Table S1b.** Treg and Teff frequency raw data. Click here for file

Additional file 2: Figure S1 Gating strategy for T_regs_ and T_effs_. Peripheral blood mononuclear cells were stained for the following markers CD3, CD4, CD25 and FoxP3. A, T_effs_ (dashed line) were defined as CD3^+^CD4^+^CD25^intermediate^ while T_regs_ (dotted line) were CD3^+^CD25^Hi^FoxP3^+^. B, FoxP3 and CD127 expression on Tregs (solid line) and Teffs (dashed line). Click here for file
