# Supplementary material for: Variability in CRP, regulatory T cells and effector T cells over time in gynaecological cancer patients: a study of potential oscillatory behaviour and correlations
Source: J Transl Med. 2014 Jun 23;12:179. doi: 10.1186/1479-5876-12-179 (PMC4082498; doi:10.1186/1479-5876-12-179)
Supplement: Additional file 1: Table S1a — CRP raw data. Table S1b. Treg and Teff frequency raw data. [file 1479-5876-12-179-S1.doc]

**Additional file 1: Table S1a: CRP raw data**

**Additional file 1: Table S1b: Treg and Teff frequency raw data**
