# Supplementary material for: Variability in CRP, regulatory T cells and effector T cells over time in gynaecological cancer patients: a study of potential oscillatory behaviour and correlations
Source: J Transl Med. 2014 Jun 23;12:179. doi: 10.1186/1479-5876-12-179 (PMC4082498; doi:10.1186/1479-5876-12-179)

**Additional file 2: Figure S1 Gating strategy for T_regs_ and T_effs_.** Peripheral blood mononuclear cells were stained for the following markers CD3, CD4, CD25 and FoxP3. A, T_effs_ (dashed line) were defined as CD3^+^CD4^+^CD25^intermediate^ while T_regs_ (dotted line) were CD3^+^CD25^Hi^FoxP3^+^. B, FoxP3 and CD127 expression on Tregs (solid line) and Teffs (dashed line).


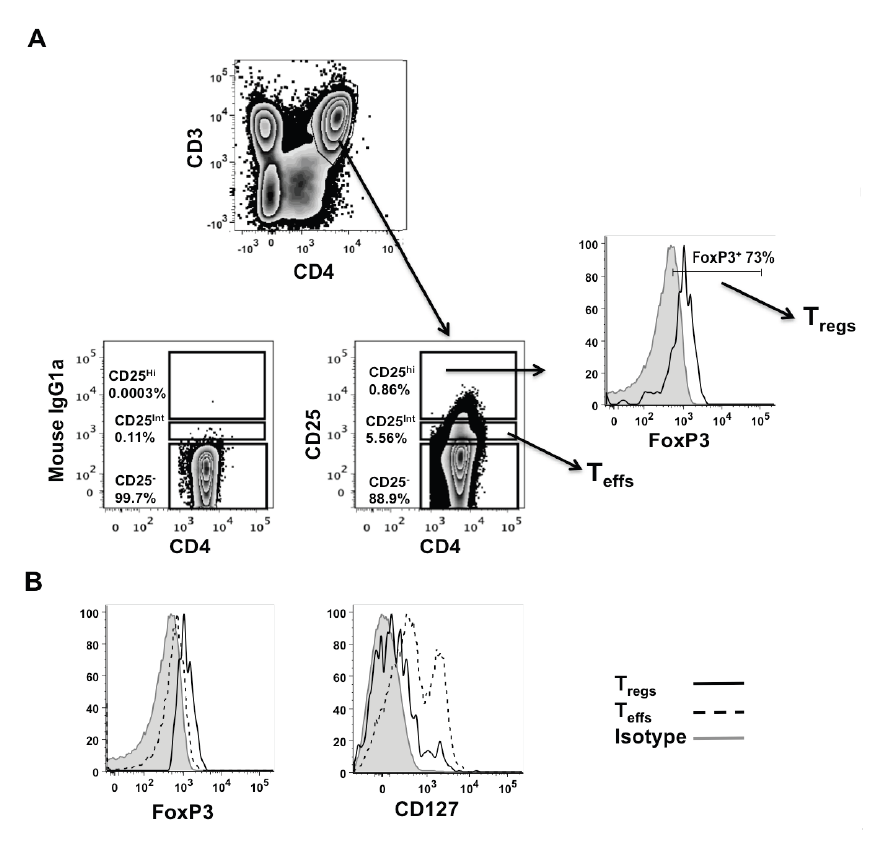

Supplement: Additional file 2: Figure S1 — Gating strategy for Tregs and Teffs. Peripheral blood mononuclear cells were stained for the following markers CD3, CD4, CD25 and FoxP3. A, Teffs (dashed line) were defined as CD3+CD4+CD25intermediate while Tregs (dotted line) were CD3+CD25HiFoxP3+. B, FoxP3 and CD127 expression on Tregs (solid line) and Teffs (dashed line). [file 1479-5876-12-179-S2.doc]
